# Chronic fatigue among healthcare staff at Farhat University Hospital Hached, Sousse, Tunisia

**DOI:** 10.1192/j.eurpsy.2026.11532

**Published:** 2026-07-31

**Authors:** R. Ghammam, H. Kalboussi, S. Ben Fredj, O. K. Ben Saad, N. Zammit, A. Skhiri, F. Chouikha, A. Erguez, R. Akrimi, W. Belguith, A. Chouchene, M. Jihene, I. Harrabi

**Affiliations:** 1 Université de Sousse, Faculté de Médecine de Sousse; 2 Hôpital Universitaire Farhat hached, Service de Médecine Préventive, LR19SP03; 3 Hôpital Universitaire Farahat Hached, Service de Médecine de Travail, LR19SP03; 4 Médecine Préventive et Communautaire, Hopital Universitaire Farhat hached; 5 Hopital Universitaire Farhat hached, Laboratoire de Biochimie; 6 Médecine de Famille, Université de Sousse, Faculté de Médecine de Sousse, Sousse, Tunisia

## Abstract

**Introduction:**

Chronic fatigue is common symptom that may affect individuals’ life. This condition is particularly concerning among health care workers and may affect their mental health and well-being.

**Objectives:**

Our study aimed to measure the proportion of fatigue chronic syndrom among health workers and its associated factors.

**Methods:**

A cross-sectional was performed among a random sample of healthcare workers at univesity Hospital Farhat Hached Sousse Tunisia. We excluded all professionals presetting severe chronic conditions. Fatigue chronic syndrome was accessed using chronic fatigue syndrome scale (FCS). Life quality was measured using SF12 questionnaire and depression using PHQ9 form.

**Results:**

Our population consisted of 205 participants, including 89.3% men. The sex ratio M/W was 0.12. Participants were 18% medical doctors, 1.5% nursing assistants, 42.4% nurses and 38% health technicians. Chronic fatigue syndrome (CFS) affected 11.2% of participants. Among health workers, 4.4% presented CSF like with insufficient fatigue and 22% chronic idiopathic syndrome. Among women 12,6% presented a CFS compared to one men (4.5%) (p=0,078). No significant association was found between CFS and night work (P=0,491). From nurses and health technicians, 12.5% presented a chronic fatigue syndrome compared to 5.4% of doctors without a significant difference (p=0.216). Physical quality of life score was significantly higher among participants with CFS (35.15±6 vs 44.56±8.75, p<0.001). The same result was found for mental quality of life. CFS group presented Higher score of depression (12.17±5.6) compared to the group with no CFS (5.43±3.92) (p<0.006). No association was observed with vitamin D level.

**Conclusions:**

Chronic fatigue syndrome is prevalent among healthcare workers, with a significant impact on both physical and mental quality of life. Its association with depression underscores the need for proactive support and screening. Although a non-significant association was observed between vitamin D levels and fatigue, further research with a larger sample size is warranted to clarify this potential link and inform targeted interventions.

**Disclosure of Interest:**

None Declared

